# A study on the cognitive-consumption behavior of sports content on OTT media platforms: applying the extended technology acceptance model (E-TAM)

**DOI:** 10.3389/fnhum.2025.1716007

**Published:** 2025-12-15

**Authors:** Yong-Seok Jang, Sun-Young Lim, Jae-Moon Lee

**Affiliations:** 1 Department of Physical Education, Kyung Hee University, Yongin-si, Gyeonggi-do, Republic of Korea; 2 Social Science Research Institute, Chung Buk National University, Cheongju-si, Chungcheongbuk-do, Republic of Korea

**Keywords:** TAM, E-TAM, OTT platform, sports content, cognitive-behavioral process

## Abstract

The purpose of this study is to provide basic data for the establishment of sustainable sports content and communication marketing strategies of effective OTT platforms by applying the extended technology acceptance model to clarify the causal relationship between the perceived usefulness, perceived ease of use, perceived enjoyment, perceived interactivity, usage intention, and usage behavior of users who watch OTT platform sports content. To achieve this purpose, 303 viewers with experience watching OTT platform sports content were used for the analysis. The data analysis methods were frequency analysis, correlation analysis, confirmatory factor analysis, and structural equation modeling analysis using SPSS 21.0 and AMOS 18.0. The results of this study are as follows. First, perceived usefulness was found to have no significant effect on usage intention. Second, perceived ease of use was found to have a significant effect on usage intention. Third, perceived enjoyment was found to have a significant effect on usage intention. Fourth, perceived interactivity was found to have a significant effect on usage intention. Fifth, usage intention was found to have a significant effect on usage behavior. Therefore, the intention of using OTT platform sports content will be to become a sustainable industry in terms of media business.

## 1 Introduction

The production and distribution methods of broadcast content are undergoing drastic changes because of the recent rapid growth of OTT platforms and structural changes in the media market (Han and Park, 2019). In the past, it was common to watch a set program for a limited time through traditional media, such as broadcasting stations; this was a passive form of consumption, providing only limited choices to viewers. However, with technological advancement and the spread of various smart media devices, such as smartphones, tablets, and smart TVs, users are able to freely select and watch their desired content whenever and wherever they want (Jang, 2022). In this context, the OTT platform originally meant a service that provided video content on the Internet through a set-top box, but recently, the concept has expanded to a nonlinear video service that allows users to freely consume content without time and space constraints on various smart devices via the Internet (Kim, 2023). As the initiative in media consumption shifts from content providers to users, the content consumption behavior of viewers also changes in an increasingly active and personalized manner.

**Figure 1:**
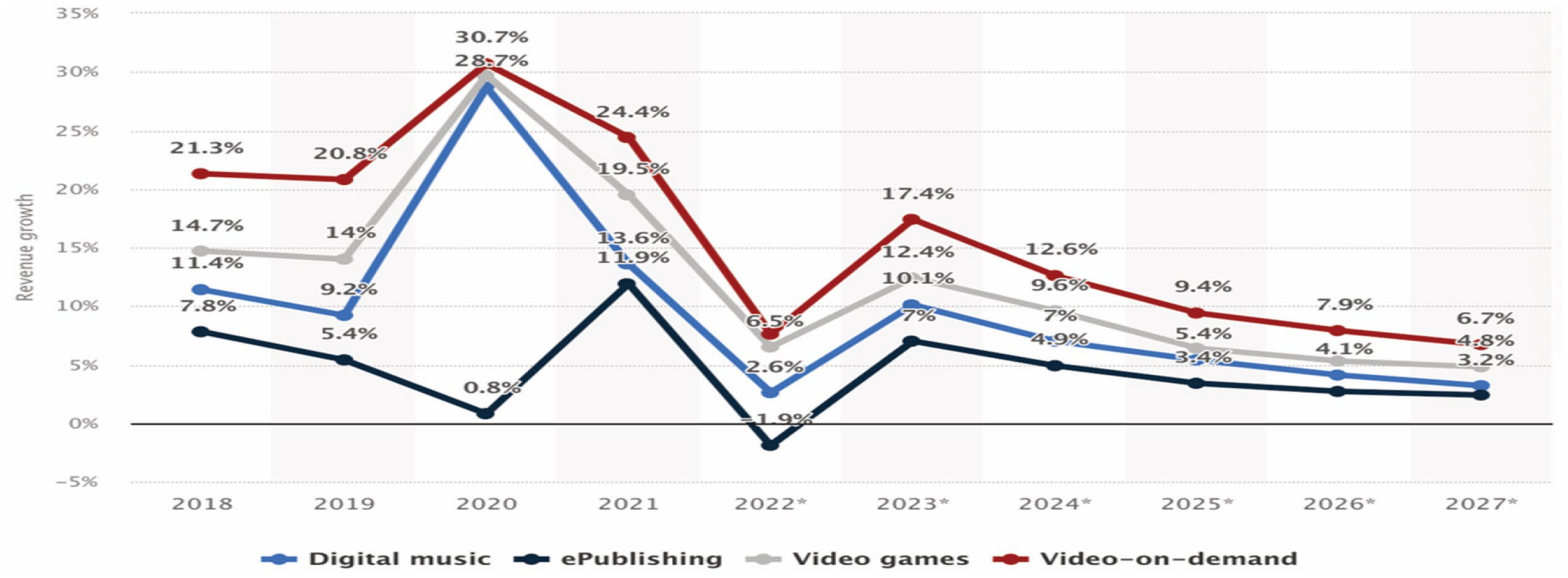
Major digital media content growth rate forecast. Reference: Statista (2022).

According to the Global Media Market Research Report, the global media and entertainment market is estimated to exceed USD 2.87 trillion by 2023, with a compound annual growth rate of approximately 5.19% from 2021 to 2026 (Statista, 2023). In 2020, the global spread of COVID-19 generally impacted the growth of the media and entertainment market negatively. However, the market actually expanded for sectors, such as OTT videos and video games, which can be easily accessed in a non-face-to-face environment, Lee (2023). In particular, the market growth rate of major global digital media content from 2018 to 2027 is estimated to be in the order of OTT video, video games, digital music, and electronic publishing. The average annual growth rate of traditional broadcasting services from 2017 to 2026 is estimated to be −0.79%, and the average annual growth rate of OTT services is estimated to be 3.93% (Figure 1).

Sports viewing offers media consumers and opportunity to engage with sports content efficiently and proactively. Furthermore, the proliferation of OTT platform services has bolstered the influx of sports video content, leading to a steady increase in sports content consumption in the new media market. Furthermore, OTT viewing is not simply content consumption; it is a continuous process of neurological, emotional, and cognitive processes. Furthermore, the brain’s reward system is activated, immersion increases attentional network and prefrontal cortex activity, and empathy and sympathy activate the amygdala and cingulate cortex. In this respect, the planning, transmission, and consumption of sports-focused content on OTT platforms are actively taking place from a cognitive neurological perspective, and the market dominance of OTT platforms is interpreted as stemming from the growth of sports media content.

Furthermore, the spread and market expansion of OTT platforms are accelerating, especially in Asia, where mobile-centric content consumption is active (Lee, 2023). This spread of OTT platform services is not only changing the consumption pattern of video content, but is also noteworthy in that it opens a new chapter in sports content consumption. As such, the influx of sports video content through OTT platforms has increased recently, and sports content consumption in the new media market continues to increase (Jang and Lee, 2021). In the past, broadcasters had exclusive broadcasting rights to sports content, which had to be viewed at a set time. Today, however, with various OTT platforms providing segments, such as live broadcasts, highlight clips, and analysis videos, accessibility for sports fans is in-creasing, and content consumption patterns are expanding. Global sports leagues and organizations are also strengthening their partnerships with OTT platforms and diversifying their content distribution by building their own streaming services or collaborating with existing platforms.

Jarvenpaa and Tractinsky (2000) likened Internet websites to offline store employees and emphasized that digital platforms are important touchpoints that can have a significant impact on corporate profits. Companies now promote their brands and products not only through the four traditional media, i.e., TV, radio, newspapers, and magazines, but also through various online platforms. Marketing competition through platforms is also very fierce. The most important factor in this environment is the quality of content. Users can easily com-pare and analyze various online platforms, and can easily ignore or not reuse systems with low content quality or insufficient user experience (Lee et al., 2021). Content quality directly affects the reliability and user loyalty of a platform and is a key consideration in establishing a company’s platform strategy.

Therefore, the intention to use according to information technology acceptance should be considered and there are various theories for evaluating website quality in relation to this. Among the theories related to information acceptance, the technology acceptance model (TAM) is the most effective model to measure users’ intention to use by applying their perceived beliefs, and examine their behaviors, such as intent to continue using and actual use (Koufaris and Hampton, 2004). TAM is an information technology acceptance theory (ITAT), developed by Davis (1986) based on the theory of reasoned action, proposed by Ajzen and Fishbein (1980). It predicts user behavior through the causal relationship between beliefs, attitudes, and behavioral intentions. TAM sets perceived usefulness and ease of use as key factors in the use of information technology and explains users’ acceptance intentions based on these factors, thereby possessing high explanatory power. However, it demonstrates certain limitations in that it does not sufficiently reflect users’ psychological complexity or social and cultural contexts (Lee et al., 2011). Accordingly, subsequent studies have pro-posed an ETAM that integrates various factors, such as perceived enjoyment and interaction as external variables of TAM. This is considered an effective approach for more precisely explaining user behavior in modern digital environments, such as OTT platforms (Agarwal and Prasad, 1997; Bhattacherjee, 2001; Davis, 1989; DeLone and McLean, 1992; Gantz and Lewis, 2014; Morris et al., 1982; Chen et al., 2002; Ryu and Seo, 2023; Lee and Lim, 2023). Ultimately, for sports content to be accepted and spread stably and continuously through the new OTT platform, it is necessary to explore and analyze the determinant factors for consumers’ usage intentions and behavior.

Therefore, the purpose of this study is to empirically analyze the structural relation-ship between technology adoption intention and the actual behavior of sports content consumers through OTT platforms by applying ETAM. Specifically, we explored the psychological factors that influence sports content users’ acceptance and continuous use of OTT platforms. To this end, in addition to perceived usefulness and ease of use, which are the main variables in the existing TAM, perceived enjoyment and interactivity, which are particularly important in the new media environment, were comprehensively considered. This is not limited to simply accepting the technology, but also includes continuous usage behavior in the analysis, thereby attempting to comprehensively view the entire cognitive, emotional, and social context of the acceptance process of OTT-based sports content. The academic contributions of this study include verification of the theoretical validity and applicability of the extended technology acceptance model in explaining sports content consumer behavior in a new media environment. The practical significance lies in establishing strategies for enhancing the competitiveness of OTT platforms in the future as well as for establishing sustainability and market expansion strategies for the sports content industry.

## 2 Theoretical background and hypothesis setting

With the rapid development of information technology and emergence of various digital platforms, the importance of research on predicting and understanding user behaviors is gradually increasing (Lee et al., 2011). In particular, in new media environments, such as OTT plat-forms, the way users accept and utilize technology shows different aspects from the existing information system usage patterns; thus, a systematic analysis of this is required.

### 2.1 The relationship between perceived usefulness of ETAM and intention to use

Perceived usefulness is based on the theory of self-efficacy and represents an individual’s belief that using a specific system will improve performance (Davis, 1989). Accordingly, Ryu (2022) found that the perceived usefulness of AI-based mobile sports positively influenced users’ intentions to continue using the service. In the same context, Tomatzky and Klein (1982) also explained that when users perceive a specific system to be more useful to use, they are more likely to use that system.

### 2.2 The relationship between perceived ease of use of ETAM and intention to use

Perceived ease of use refers to the extent to which an individual believes that he or she will have no difficulty using a particular information technology or system (Davis, 1989). In other words, it refers to the extent to which an individual believes that it is easy to accept and use information technology or innovative products (Chen et al., 2002). In this regard, Sun and Zhang (2006) argued that the easier it is to use a new technology or system, the higher the rate of its usage. Seong and Hong (2022) stated that a technology or system with high perceived ease of use can reduce the user’s effort and produce higher performance with the same amount of effort, so the more convenient it is to use a new technology or system, the more positive demand intention is formed.

### 2.3 The relationship between perceived enjoyment of ETAM and intention to use

Perceived enjoyment refers to the enjoyment, fun, and interest experienced by users when consuming sports content through OTT platforms and acts as a key factor in accepting sports content (Rauschnabel et al., 2017; Venkatesh, 2000). Sports are inherently hedonic in nature and enjoyment positively impacts technology acceptance (Faqih, 2022). In other words, since the element of enjoyment is one of the hedonic benefits that users experience when enjoying sports content, if they perceive sports content provided through various OTT platforms as enjoyable, they are more likely to choose, or continuously use that technology (Ryu, 2022; Sun and Zhang, 2006; Anandarajan et al., 2000).

### 2.4 The relationship between perceived interactivity of ETAM and intention to use

Perceived interactivity refers to the degree to which participants in a communication process control each other’s interactions and enable role exchanges (Rogers, 1986; Oh and Sundar, 2015). In this regard, Liu and Shrum (2002) described interaction as a complex process, similar to cognitive events or psychological behaviors; it is the extent of influence of two or more communication participants on each other’s medium or message, and of the simultaneous occurrence of this interaction. Kiousis (2002) defined interactivity as the dynamics of interaction and information exchange between participants and emphasized that it significantly impacts user participation. Thus, interactivity is an important factor that can induce trust, satisfaction, and continuous use of sports content by consumers regarding the technological acceptance of OTT platform services (Cha, 2019).

### 2.5 The relationship between intention and behavior to use

Intention to use, which is a prerequisite for actual behavior, involves concretizing one’s beliefs (Venkatesh and Bala, 2008). According to Azizi et al. (2020), the higher the specific intention of an individual, the stronger the desire to act, which is more likely to lead to actual behavior. In the same context, Azizi et al. (2020) also presented research results showing a high correlation between the intention and behavior to use technology. In summary, analyzing usage intentions can increase the predictive power of actual usage behaviors (Midgley and Dowling, 1978).

### 2.6 Hypothesis setting and research model

**Figure 2:**
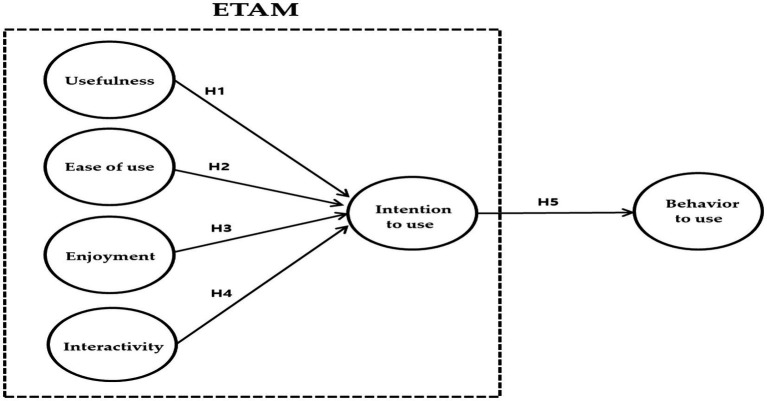
Research model.

We established the following hypotheses and models based on the results of previous studies (Figure 2).

*H1*: The perceived usefulness, a sub-factor of ETAM, will significantly affect the intention to use.

*H2*: The perceived ease of use, a sub-factor of ETAM, will significantly affect the intention to use.

*H3*: The perceived enjoyment, a sub-factor of ETAM, will significantly affect the intention to use.

*H4*: The perceived interactivity, a sub-factor of ETAM, will significantly affect the intention to use.

*H5*: The intention to use will significantly affect the behavior to use.

## 3 Methods

### 3.1 Participants

To select research subjects that fit the purpose of this study and secure the representativeness of the sample, this study conducted a limited survey targeting domestic users aged 19 years or older, who are citizens of the Republic of Korea and have experience watching sports content through OTT platforms. Among the non-probability sampling methods (random sampling method), the judgment sampling method was used to select research participants satisfying the above conditions. Judgmental sampling is suitable for this study because it allows researchers to select respondents appropriate for research purposes, thereby obtaining more in-depth and meaningful information from subjects with specific knowledge or experience (Palinkas et al., 2015). Data were collected via an online survey. The questionnaire was created using Google Forms, and participants voluntarily responded by accessing the link provided by the researcher. Online surveys have the advantage of collecting data without revealing the identity of respondents, which can lead to honest responses to sensitive topics (Joinson, 1999). Additionally, because of no geographical restrictions, surveys can be conducted on wider sample groups (Evans and Mathur, 2005). These characteristics make online surveys an effective research method, especially in a modern society that is accustomed to the digital environment (Dillman et al., 2014). The online survey was conducted for 3 months from January 1, 2025, to April 1, 2025, and 315 questionnaires were sampled and returned. Of these, 12 with in-sincere or no responses were excluded, and data from 303 questionnaires were used for the final analysis (Table 1).

**Table 1:** Demographic characteristics of participants.

Variables	Classification	Frequency (n)	Percentage (%)
Gender	Male	153	50.5
	Female	150	49.5
Age	20 s	197	65.0
	30 s	47	15.5
	over 40 s	59	19.5
Sports content types used	Less than 30 min	17	5.6
	More than 30 min-less than 1 h	73	24.1
	More than 1 h-less than 2 h	117	38.6
	More than 2 h-less than 3 h	52	17.2
	More than 3 h	44	14.5
Sports content time used	Soccer	159	52.5
	Baseball	73	24.1
	Basketball	31	10.2
	Volleyball	25	8.3
	e-Sports	15	4.9
Total		303	100

Above all, this study did not collect or record sensitive information on the research subjects, as suggested by Public Institutional Ethics Committee Act on Bioethics and Safety (2013), therefore, deliberation by the Korean Institutional Review Board was unnecessary. The above information was stated in the research subject description section on the first page of the online questionnaire, and the survey was conducted after obtaining prior consent from all research subjects.

### 3.2 Measurement tool

In this study, a questionnaire was developed to examine the influence of the four sub-perceptual variables of the existing TAM model’s perceived usefulness and ease of use and the added perceived enjoyment and interactivity of the ETAM model on the usage behavior of OTT platform sports content. To this end, based on the studies of Davis (1989), Davis et al. (1992), and Moon and Kim (2001), the questionnaire was modified and supplemented to fit the purpose of this study based on the items used by Lee et al. (2016), Ryu and Seo (2023), and Lee and Lim (2023) in Korea. The final questionnaire consisted of 26 items, specifically with four items each on perceived usefulness, ease of use, enjoyment, intention to use, and demographic characteristics (gender, age, sports content types used, sports content time used), and three items each on interactivity and usage behavior. Finally, the questionnaire was completed by the respondents using the self-assessment method, and all questions, except for the participants’ general characteristics, were measured using a 5-point Likert scale (1 = not at all, 5 = strongly agree).

### 3.3 Validity and reliability of measurement

Expert groups of professors (PhDs) in sports management verified the convergent validity of the measurement tools (questionnaires) used in this study. A confirmatory factor analysis (CFA) was performed to assess discriminant validity (Table 2).

**Table 2:** Confirmatory factor analysis.

Variables	Factors	*SC*	*SE*	*T*	C. R	AVE	Cronbach’s α
Perceived usefulness	Usefulness of navigation	0.726	–	–	0.852	0.594	0.847
	Usefulness of information provided	0.898	0.077	13.016			
	Usefulness of overall use	0.688	0.087	10.817			
	Usefulness of rapid use	0.663	0.090	10.302			
Perceived ease of use	Ease of use	0.777	–	–	0.850	0.587	0.899
	Ease of navigation	0.662	0.071	13.013			
	Ease of acquisition	0.785	0.089	11.055			
	Understandability of navigation	0.642	0.080	9.877			
Perceived enjoyment	Pleasure of use	0.785	–	–	0.877	0.642	0.701
	Happiness of use	0.775	0.089	10.989			
	Interest in use	0.642	0.123	10.556			
	Positivity of use	0.757	0.089	10.658			
Perceived interactivity	Interaction with others	0.811	–	–	0.910	0.771	0.884
	Social relationships	0.875	0.031	20.111			
	Information exchange	0.840	0.035	15.640			
Intention to use	Intention to reuse	0.633	–	–	0.907	0.714	0.893
	Intention to continue using	0.755	0.123	11.120			
	Intention to recommend	0.951	0.156	13.555			
	Intention to positively recommend	0.815	0.175	14.545			
Behavior to use	Continuous use behavior	0.622	–	–	0.898	0.749	0.886
	Reuse behavior	0.752	0.154	11.454			
	Recommended use behavior	0.903	0.199	13.444			

*χ*^**2**^ = 341.246 (df = 118, *p* = 0.000), *Q* = 2.689, NFI = 0.856, TLI = 0.904, CFI = 0.912, RMR = 0.051, and RMSEA = 0.071.

The CFA analysis results were *χ*^**2**^ = 341.246 (*df* = 113, *p* = 0.000), Q value = (*χ*^**2**^/*df*) was 2.689, NFI = 0.856, TLI = 0.904, CFI = 0.912, RMR = 0.051, and RMSEA = 0.071. Since the *χ*^**2**^ value is determined by the value of [*F* × (sample size −1)], it is sensitive to the sample size, so even for the same model, rejection is determined by the sample size, making it difficult to properly evaluate the model itself (Kim et al., 2009). The Q value is used as an alternative to these problems; if the Q value is 3 or less, the model can be judged as suitable (Wheaton et al., 1977). Additionally, Bagozzi and Dholakia (2002) evaluated that a model is suitable when NFI and CFI are 0.8 to 0.9 or higher, and RMR and RMSEA are 0.05 or 0.08 or lower. Accordingly, the results of the overall confirmatory factor analysis showed that these parts were relatively satisfactory, and overall, there was no problem in judging the fit index. The convergent validity of each variable, construct reliability (CR), and the average variance extracted (AVE) were calculated. The analysis revealed that the concept reliability of all variables was 0.587–0.749, and AVE was 0.850–0.980, satisfying the values of conceptual reliability (CR) of 0.7 or higher and AVE of 0.5 or higher as suggested by Hair et al. (2006), indicating that each variable demonstrates convergent validity. Next, to evaluate the reliability of the scale used in this study, an internal consistency reliability analysis method using Cronbach’s *α* coefficient was used, and the results of the analysis, using the theory of Nunnally and Bernstein (1994), revealed that the Cronbach’s α value must be 0.8 or higher.

## 4 Results

### 4.1 Correlation analysis

Correlation analysis was performed to check for correlations and multicollinearity between the variables. The higher the value of the correlation coefficient, the stronger the relationship between the two variables, causing multicollinearity problems and reducing the validity of the measurement tool. Accordingly, Challagalla and Shervani (1996) described multicollinearity as when one independent variable is well predicted by several other variables and stated that this occurs when the value of the correlation coefficient is 0.8 or higher. After conducting a correlation analysis to identify this problem, it was observed that there was no multicollinearity problem because no variables showed a correlation of more than 0.8 (Table 3). Therefore, it can be seen that there is no problem in structurally verifying the theoretical model of this study.

**Table 3:** Correlation analysis.

Variables	1	2	3	4	5	6
Perceived usefulness^1^	1					
Perceived ease of use^2^	0.477**	1				
Perceived enjoyment^3^	0.222**	0.259**	1			
Perceived interactivity^4^	0.175**	0.127**	0.364**	1		
Intention to use^5^	0.243**	0.503**	0.519**	0.358**	1	
Behavior to use^6^	0.176**	0.373**	0.494**	0.320**	0.769**	1

**p < 0.01.

1 Perceived usefulness.

2 Perceived ease of use.

3 Perceived enjoyment.

4 Perceived interactivity.

5 Intention to use.

6 Behavior to use.

### 4.2 Model verification

The results of the analysis verified the suitability of the structural model established in this study, which is as follows: *χ*^**2**^ = 341.246, df = 118, NFI = 0.856, TLI = 0.904, CFI = 0.912, RMR = 0.051, RMSEA = 0.071 (Table 4).

**Table 4:** Fit index of the research model.

A construct	*χ^2^*	*df*	*p*	NFI	TLI	CFI	RMR	RMSEA
Acceptance level	341.246	118	0.000	0.856	0.904	0.912	0.051	0.071
Acceptance criteria	–	–	–	More than 0.8 ~ 0.9	More than 0.8 ~ 0.9	More than 0.8 ~ 0.9	Less than 0.05 ~ 0.08	Less than 0.05 ~ 0.08

This is the same figure as the confirmatory factor analysis fit, because confirmatory factor analysis, wherein all relationships between latent variables are set as correlations, and structural equation modeling, wherein all relationships between latent variables are set as correlations or causal relationships, are equivalent models (Yoo, 2012). Therefore, there were no statistical errors in determining the model fit indices for each variable.

### 4.3 Hypothesis testing

The structural equation model was used to confirm the causal relationship between the hypotheses established in this study and the research model variables (Table 5).

**Table 5:** Hypothesis testing result.

H	Path	*SE*	*CR*	*p*	Accept/Reject
H1 Perceived usefulness → intention to use	−0.070	0.059	−1.402	0.162	Reject
H2 Perceived ease of use → intention to use	0.420	0.055	8.439	0.000 ***	Accept
H3 Perceived enjoyment → intention to use	0.358	0.038	7.521	0.000 ***	Accept
H4 Perceived interactivity → intention to use	0.186	0.046	4.003	0.000 ***	Accept
H5 Intention to use → behavior to use	0.769	0.040	20.849	0.000 ***	Accept

***p < 0.001.

The results of the hypothesis testing analysis indicated the following: First, the path coefficient of H1 was −0.070; hence, H1 was rejected. Second, the path coefficient of H2 was 0.420 (*t* = 8.439, *p* < 0.001); thus, H2 was supported. Third, the path coefficient of H3 was 0.358 (*t* = 7.521, *p* < 0.001); therefore, H3 was accepted. Fourth, the path coefficient of H4 was 0.186 (*t* = 4.003, *p* < 0.001), thereby supporting H4. Finally, the path coefficient of H5 is 0.769 (*t* = 20.849, *p* < 0.001), thus supporting H5.

## 5 Discussion

This study empirically analyzed the structural relationship between the intention to adopt technology and the actual behavior of sports content consumers through OTT plat-forms by applying ETAM. It identified the psychological factors that affect sports content users’ adoption and continuous use of OTT platforms. Based on the results, the following conclusions were drawn:

First, it was confirmed that perceived usefulness, a subfactor of ETAM, did not have a significant effect on intention to use; thus, H1 was rejected. This result conflicts with those of previous studies. Thus, the following implications were derived. DeLone and McLean (1992) presented the usefulness of information quality as one of the key success factors of website quality characteristics through an information system success model, and argued that the usefulness of information closely influences the intention to use. Similarly, Igbaria and Livari (1995) and Gefen and Straub (1997) reported that perceived usefulness positively affects user value and intention to use. Lin and Lu (2001) also presented results showing that the perceived usefulness and ease of use of a website significantly affect the intention to use it. These conflicting results can be interpreted as being due to the differences in the characteristics of the content being studied. While previous studies have mostly targeted administrative information systems or general information services, this study targeted sports content, which possesses special properties, such as playfulness, dramatic narrative structure, and unpredictability of results. The reason why perceived usefulness did not significantly influence usage intention is that users of OTT platform sports content tend to prioritize enjoyment and immersion over utility. This suggests that entertainment experiences and “playfulness” better explain usage intention than utilitarian tools. Furthermore, in modern society, where universal technologies are already widespread and commonplace, and technologies such as OTT and SNS YouTube have become part of everyday life, the influence of usefulness is likely to have weakened. Furthermore, the existence of alternative services may lead to many similar platforms, which may reduce differentiation in usefulness. Emotional and social factors may also be important factors, and social and emotional factors such as friend recommendations, popular trends, and athlete preferences are judged to have a greater influence on usage intention. This suggests that the perceived usefulness of the subject of this study did not significantly influence usage intention on OTT platform sports content. Sports content users are more likely to respond sensitively to factors, such as immediate immersion, emotional response, and real time interactivity, than to the usefulness of information, which may be the background for the fact that perceived usefulness is not directly linked to usage intention. Nonetheless, studies, such as Ryu (2022) and Tomatzky and Klein (1982), demonstrate that perceived use-fulness is still a key factor in usage intention, suggesting that the results of this study do not completely deny the importance of perceived usefulness. Therefore, to effectively im-prove the perceived usefulness of sports content on future OTT platforms, efforts are needed to quickly and intuitively provide information, such as game schedules, player in-formation, highlight videos, and live broadcast links, through user-centered user interfaces (UI) and user experiences (UX). Furthermore, improving users’ information utilization experience by improving information accessibility and system navigation convenience indirectly influences usage intention.

Second, perceived ease of use, a sub-factor of ETAM, was found to significantly affect intention to use; therefore, H2 was accepted. These results suggest that perceived ease of use is a key variable influencing the usage intention of OTT platform sports content users. In other words, when sports content users perceive OTT services as easy to use, their usage intention increases. This is consistent with the results of many previous studies that show that perceived ease of use significantly affects the intention to use, thereby theoretically supporting the results of this study (Chang et al., 2019; Arenas-Gaitán et al., 2015; Sun and Zhang, 2006; Seong and Hong, 2022). Accordingly, OTT platform sports con-tent providers need to take a strategic approach to improve user convenience when building their platforms. For example, designing a UI that allows users to compare and select key content on one screen without having to repeatedly navigate through all pages within the platform, or improving the search function so that users can quickly locate the desired information, are good examples. Additionally, quickly updating sports-related information, such as match schedules, real-time results, and highlights, can increase the convenience of information access, contributing to a positive perception of the platform.

Third, perceived enjoyment, a sub-factor of ETAM, was found to have a significant effect on the intention to use; therefore, H3 was accepted. These results show a direction consistent with several previous studies (Ryu, 2022; Sun and Zhang, 2006; Rauschnabel et al., 2017; Venkatesh, 2000; Anandarajan et al., 2000; Igbaria and Livari, 1995). Yoon (2002) emphasizes that even with excellent quality and service of websites and digital platforms, user churn may occur if the content lacks interest and enjoyment. This suggests that emotional elements of user experience can more decisively influence platform use than simple technical excellence, supporting the need for OTT platform sports content providers to enhance the excitement and immersion of their content. In particular, sports content can induce a high level of enjoyment by combining the real-time nature of the game, unpredictability, and emotional immersion in the team and players. Therefore, the excitement of the content itself can be seen as playing a decisive role in users’ intention to continue using it. Moon and Kim (2001) attempted to explain an individual’s intrinsic motivation to accept information technology by adding perceived enjoyment to the existing TAM, and the results showed that enjoyment significantly affected the intention to use. This is consistent with the results of this study and confirms that enjoyment is not only a secondary factor, but also a core motivation for acceptance. Thus, in a situation where OTT platforms must provide differentiated user experiences in a highly competitive market structure, strategies to maximize the enjoyment of sports content are even more important. Therefore, platform providers must provide enjoyment to users through content and service compositions, such as pop-ular sports broadcasts, live coverage of various events, user-participatory content (e.g., quizzes, real-time voting, and fan communities), and personalized recommendations. Ad-ditionally, content that combines events, reward programs, and emotional production can increase users’ immersion and ultimately strengthen their intention to continue using the service.

Fourth, perceived interactivity, a sub-factor of ETAM, was found to have a significant effect on the intention to use; therefore, H4 was accepted. Hoffman and Novak (1996) explain perceived interactivity as an antecedent of usage intention and behavior, while Kang et al. (2006) emphasize social influence and perceived interactivity as factors that influence mobile Internet use. Further, Ahn et al. (2009) report in a study, based on the extended technology acceptance model, that perceived interactivity significantly affected the intention to use Internet brand community sites, similar to the results of this study (Liu and Shrum, 2002; Kiousis, 2002; Cha, 2019). This can be interpreted as the result of the content consumption experience on OTT platforms expanding beyond simple passive viewing to active interactions between users. In particular, because sports content induces active communication between view-ers, such as cheering, exchanging opinions, and real-time reactions, interactivity plays an even more important role in the context of content consumption. Therefore, OTT platform sports content providers must actively integrate functions that can induce active interactions between users in their systems. For example, interactivity can be enhanced by providing real-time chat windows, user voting during games, fan community forums, and user-generated content (UGC) sharing spaces. These functions can ultimately contribute to strengthening the attachment to the platform and the intention to continue using it by in-creasing emotional bonds and engagement between users.

Finally, the intention to use significantly affects the behavior to use; thus, H5 is accepted. Davis (1989) emphasize that the intention to use is a key variable explaining actual behavior in the technology acceptance model, and suggested its preceding role in under-standing the acceptance of the system. The results of this study support this claim (Azizi et al., 2020; Raman and Don, 2013) and show that the intention to use has strong explanatory power, leading to actual behavior in the OTT platform sports content environment. Additionally, Agarwal and Prasad (1997) emphasize that human behavior cannot occur without intention and that intention is the most immediate determinant of actual behavior, suggesting a logical structure that is consistent with the results of this study. Accordingly, OTT platform sports content providers should enhance usage intention by increasing users’ perceptions of ease of use, usefulness, enjoyment, and interactivity, which can ultimately lead to an increase in actual usage behavior. For example, providing a user-friendly interface, composing useful and immersive sports content, and strengthening communication between users can enhance the intention to use, leading to actual usage behavior.

## 6 Limitations of the research

This study empirically clarified the factors affecting the usage intention and actual usage behavior of OTT platform sports content users based on ETAM, and derived theoretical and practical implications through this. However, there are some limitations in the research process, and we intend to suggest directions for follow-up research to supplement the. First, there were limitations in the selection of research subjects. This study con-ducted a limited survey targeting citizens in the Republic of Korea, aged 19 years or older, who had experience watching sports content on OTT platforms. Therefore, there may be errors in generalizing the results of this study to an entire user group with various cultural backgrounds. Future studies should expand the sample nationwide or use various regions, age groups, and usage characteristics to conduct analyses that consider representative-ness and generality. Second, this study comprehensively analyzed the entire sports con-tent of OTT platforms; however, user experience and acceptance behavior may differ de-pending on the service characteristics and operating strategies of each platform. Therefore, future research may be able to derive more in-depth and precise results by conducting limited analyses that focus on specific OTT platforms. Third, this study conducted the analysis using mainly quantitative research methods; however, if a qualitative approach to user perceptions and behaviors is applied in parallel, a richer and more in-depth interpretation will be possible. In future studies, qualitative methodologies, such as in-depth interviews and focus group interviews (FGI), can be used in parallel to capture the diversity and con-text of users’ experiences more precisely. Sports content is rich in visual and auditory stimuli, such as rapid scene transitions, player movements, and commentary intonation, which strongly induces viewer attention. From a consumer neuroscience perspective, this is manifested by increased activity in the frontal and parietal lobes, allowing viewers to selectively focus their attention on key moments in the game. This attentional mechanism plays a crucial role in determining content immersion and information processing efficiency. When watching sports media, viewers tend to experience the player’s actions and emotions “imitatively.” Neuroscientifically, this is linked to the activation of mirror neurons, and viewers’ emotions are particularly aligned with those of the player during scoring scenes, injury scenes, and moments of decisive victory or defeat. This empathy is confirmed by increased activity in the amygdala and anterior cingulate cortex, ultimately serving as a key mechanism for viewers to psychologically identify with a specific team or player. Therefore, we applied the E-TAM model to analyze the relationship between various variables in terms of cognitive neuroscience and consumer psychological behavior of sports consumers on OTT platforms. This study will provide further insight into the cognitive science and marketing psychology of OTT platform media consumers and will be a step forward.

Finally, because sports content has unique characteristics, such as the real-time nature of the game, competitiveness, and emotional immersion, unlike other genres of content, future research will be able to identify users’ segmented acceptance behaviors and establish appropriate strategies through analysis by type of sport (e.g., soccer, baseball, e-sports) or viewing purpose.

## 7 Conclusion

This study aims to explain the sustainable usage behavior of OTT platform sports content users by applying ETAM. To this end, we analyzed the direct effects of perceived usefulness, ease of use, enjoyment, and interactivity on usage intention, and empirically verified the path from usage intention to actual usage behavior. The following conclusions were drawn from the results: First, perceived usefulness does not significantly affect usage intention, suggesting that emotional satisfaction and experience are more important than informational value for sports content consumption. Second, perceived ease of use positively affects usage intention, indicating that content accessibility and usability strengthen platform usage intention. Third, perceived enjoyment significantly affects usage intention, confirming that emotional immersion and enjoyment are important determinants of sports content consumption. Fourth, perceived interactivity positively affects usage intention, indicating that communication and participation between users contribute to intention formation. Finally, usage intention significantly affects actual behavior to use, empirically proving that the core path of TAM, ‘intention to use→behavior to use,’ is valid in the OTT platform environment.

## Data Availability

The original contributions presented in the study are included in the article/supplementary material, further inquiries can be directed to the corresponding authors.
